# Exploring Molecular Targets for Mitochondrial Therapies in Neurodegenerative Diseases

**DOI:** 10.3390/ijms241512486

**Published:** 2023-08-06

**Authors:** Germán Plascencia-Villa, George Perry

**Affiliations:** Department of Neuroscience, Developmental and Regenerative Biology, The University of Texas at San Antonio (UTSA), San Antonio, TX 78249, USA; george.perry@utsa.edu

**Keywords:** Alzheimer’s disease, Parkinson’s disease, neurodegeneration, mitochondria, mitochondrial dysfunction, oxidative stress, neurons

## Abstract

The progressive deterioration of function and structure of brain cells in neurodegenerative diseases is accompanied by mitochondrial dysfunction, affecting cellular metabolism, intracellular signaling, cell differentiation, morphogenesis, and the activation of programmed cell death. However, most of the efforts to develop therapies for Alzheimer’s and Parkinson’s disease have focused on restoring or maintaining the neurotransmitters in affected neurons, removing abnormal protein aggregates through immunotherapies, or simply treating symptomatology. However, none of these approaches to treating neurodegeneration can stop or reverse the disease other than by helping to maintain mental function and manage behavioral symptoms. Here, we discuss alternative molecular targets for neurodegeneration treatments that focus on mitochondrial functions, including regulation of calcium ion (Ca^2+^) transport, protein modification, regulation of glucose metabolism, antioxidants, metal chelators, vitamin supplementation, and mitochondrial transference to compromised neurons. After pre-clinical evaluation and studies in animal models, some of these therapeutic compounds have advanced to clinical trials and are expected to have positive outcomes in subjects with neurodegeneration. These mitochondria-targeted therapeutic agents are an alternative to established or conventional molecular targets that have shown limited effectiveness in treating neurodegenerative diseases.

## 1. Introduction

Neurodegenerative diseases are a heterogeneous group of neurological disorders caused by the progressive deterioration of function or structure of the nervous system cells in the brain or peripheral nervous system. Neurodegeneration affects a considerable number of subjects, mostly older individuals (>65 years old), showing a progressive deterioration in cognition, memory, and/or motor functions. Neurodegenerative diseases share histopathological hallmarks, including abnormal protein aggregation, synaptic and neuronal network dysfunction, altered proteostasis, cytoskeletal abnormalities, altered energy metabolism, DNA/RNA defects, neuroinflammation, and progressive neuronal death [1].

**Figure 1:**
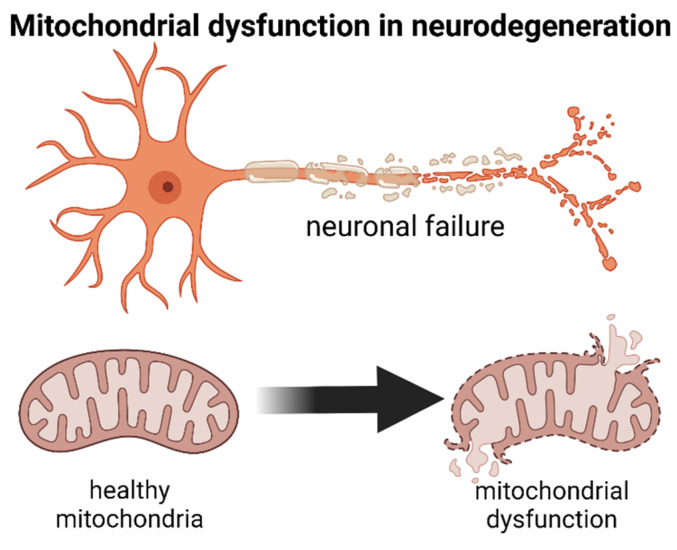
Neurodegeneration is characterized by mitochondrial dysfunction.

Alzheimer’s disease (AD) and Parkinson’s disease (PD) are the most common neurodegenerative diseases. More than 6.2 million people in the USA may have AD, and around 1 million people are affected by PD. AD is characterized by a progressive accumulation of amyloid-β (Aβ) forming plaques extracellularly and neurofibrillary tangles of tau located inside neurons [2]. Further neuropathological changes in the brain regions affected by AD include neuroinflammation, synaptic failure, oxidative stress, mitochondrial dysfunction, and progressive neuronal death [3]. Mitochondrial dysfunction in AD is linked to impaired energy metabolism, increased oxidative stress, alterations in mtDNA, abnormal dynamics in mitochondrial fusion-fission, deficiencies in axonal trafficking, and impaired mitochondrial biogenesis, impacting endoplasmic reticulum functions, alterations in proteostasis, and impaired mitophagy [4]. Figure 1.

The progressive neuronal loss in the substantia nigra (midbrain dopaminergic nucleus) causes dopamine deficiency in PD, and the aggregation of α-synuclein in Lewy bodies stands as its main neuropathological hallmark [5]. As a consequence, PD subjects commonly present alterations in motor movement and reward functions. The underlying mechanisms behind PD pathology include α-synuclein, oxidative stress, alterations in calcium homeostasis, axonal transport, neuroinflammation, and mitochondrial dysfunction (Figure 1). The roles of mitochondrial dysfunction as an initiator, propagator, or bystander in PD still remain poorly understood [6], but evidence points to mutations in the genes of putative kinase 1 (*PINK1*) and parkin (*PRKN*) in mediating mitochondrial damage and mitophagy and disruption of the subunits of mitochondrial complex I (MCI) [7]. Furthermore, the genes *SNCS*, *LRRK2,* and *CHCHD2* are also associated with mitochondrial dysfunction in PD.

Decades of basic, translational, and clinical research have identified eight main hallmarks present in neurodegenerative diseases: pathological protein aggregation, synaptic and neuronal network dysfunction, aberrant proteostasis, cytoskeletal abnormalities, altered energy homeostasis (mitochondrial dysfunction), DNA/RNA defects, inflammation, and neuronal cell death [1]. The high activity and energy consumption make neurons susceptible to defects in mitochondrial functions and energy metabolism. Here, we present relevant therapeutic approaches that target mitochondrial functions as promising alternatives to conventional treatments for neurodegenerative diseases.

## 2. Conventional Targets for Therapies in Neurodegeneration

Only four small-drug compounds are approved by the US FDA to treat symptoms of AD, as are two immunotherapies that target amyloid-β deposits [8]. These small drugs have two different mechanisms of action, such as cholinesterase (AChE) inhibitors or *N*-methyl D-aspartate (NMDA) antagonists. Donepezil, rivastigmine, and galantamine are AChE inhibitors administered to treat mild to moderate AD, whereas memantine is an NMDA antagonist directed at treating moderate to severe AD. Overall, these small-drug compounds aim to increase the amount of neurotransmitters in cholinergic synapses to treat the progression of cognitive decline, preserving thinking ability and memory loss in AD patients. In contrast, aducanumab (commercial name: Aduhelm) is an immunotherapy designed to target Aβ aggregates to promote their immune clearance from affected areas of the brain. The second therapeutic antibody, lecanemab (commercial name: Leqembi), received FDA approval via the Accelerated Approval process in January 2023. This humanized IgG1 monoclonal antibody recognizes the Aβ soluble protofibrils with high affinity to reduce amyloid deposits in early AD [9].

However, the clinical benefits of these immunotherapies have been questioned, as have some concerns regarding side effects such as amyloid-related imaging abnormalities, infusion-related reactions, edema, life-threatening micro-hemorrhages or effusions, and the extremely overpriced treatment course suggested for AD subjects [10,11]. Overall, the amyloid-targeted therapies have failed to show clinical benefit in clinical trials in patients with mild-to-moderate AD; these clinical observations indicate the possibility that Aβ deposition represents an epiphenomenon or response rather than a primer cause of AD [12].

**Figure 2:**
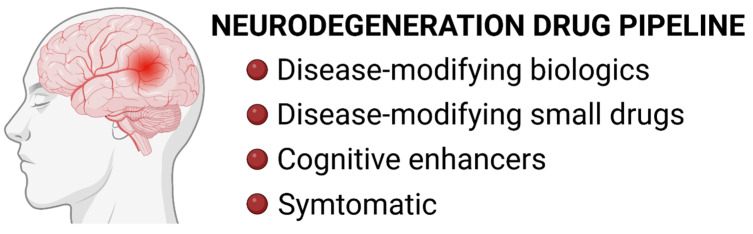
Neurodegeneration drug pipeline. Therapeutic compounds for the treatment of neurodegeneration (AD or PD) are classified into four main categories: (1) Disease-modifying biologics (i.e., therapeutic antibodies), (2) Disease-modifying small drugs, (3) Cognitive enhancers, and (4) Symptomatic drugs.

The therapeutic drugs in current development for the treatment of AD include 143 agents evaluated in 172 clinical trials [13]. The AD drug pipeline includes 31 compounds in phase 3 clinical trials, 82 compounds in phase 2, and 30 more compounds in phase 1. By organizing these compounds by the mechanism of action, 119 of these compounds are disease-modifying (40 biologics and 79 small drug molecules), 14 are cognitive enhancers, and 10 drugs are intended for symptoms (Figure 2). The primary molecular targets of these treatments are amyloid-β, tau, inflammation, and synaptic plasticity/neuroprotection. Mitochondrial dysfunction is considered or mentioned as the mechanism of action of only three drugs (blarcamesine, tricaprilin, and metabolic cofactor supplements).

In the case of Parkinson’s disease, levodopa (*L*-dopa) and carbidopa are two of the most commonly prescribed medications to control symptoms. These small-drug compounds are precursors of the neurotransmitter dopamine. Additionally, other compounds with different mechanisms of action are also approved for the treatment of PD. This includes catechol-O-methyltransferase inhibitors (entacapone, tolcapone, and opicapone), dopamine agonists (pramipexole, ropinirole, apomorphine, and rotigotine), monoamine oxidase inhibitors (selegiline, rasagiline, and safinamide), NMDA antagonists (amantadine), adenosine 2A antagonist (istradefylline), and anticholinergic (trihexyphenidyl and benztropine). As described, all these small drugs aim to restore the brain’s dopamine levels and reduce motion symptoms in PD patients, but unfortunately, all these compounds present some side effects, such as nausea, dizziness, anxiety, somnolence, and digestive problems, among others.

The efforts for the development of novel PD therapies include 147 compounds in clinical trials [14]. Among these are 91 symptomatic treatments and 56 disease-modifying treatments (Figure 2). Only 22 compounds have advanced to phase III; 74 drugs are currently in phase II, and the remaining 51 are in phase I. Overall, the majority of the PD therapeutic compounds are intended or designed for dopaminergic activity or symptom relief, and only three of the 147 therapeutic drugs target mitochondrial activity as a mechanism of action (UDCA, nicotinamide riboside, and terazosin). However, some PD therapies are classified as antioxidants or inhibitors that indirectly impact the mitochondrial activity of neurons; some of these will be discussed below.

## 3. Alternative Molecular Targets for Neurodegeneration: Mitochondrial Therapies

**Figure 3:**
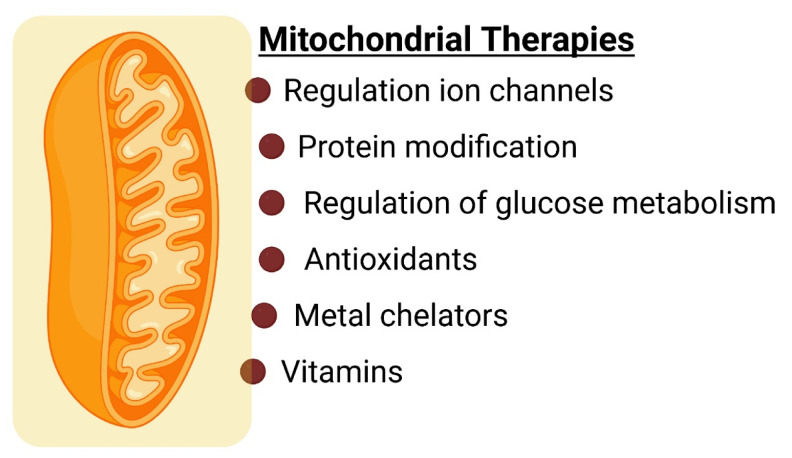
Mitochondrial therapies for neurological disorders.

Neurodegeneration is characterized by a direct or indirect impairment of mitochondrial functions, leading to low ATP availability, neuronal alterations, synaptic dysfunction, alterations in calcium homeostasis, cytoskeletal dynamics, and proteostasis [1]. Additionally, mitochondrial dysfunction is linked to oxidative damage, molecular dysfunction, and progressive neuronal death. Despite their central role in human health and disease, there are no approved therapeutic drugs that directly target mitochondria [15]. Alternative molecular targets in mitochondria include regulation of calcium ion (Ca^2+^) transport, protein modification, regulation of glucose metabolism, antioxidants, metal chelators, and vitamin supplementation (Figure 3). These therapeutic compounds aim to re-establish or preserve mitochondrial activity and restore the balance of the antioxidant systems in the neurons.

There are three therapeutic agents for AD in phase III clinical trials with a mechanism of action related to metabolism/bioenergetics: metformin, semaglutide, and tricaprilin. Moreover, the agents focusing on oxidative stress are hydralazine, icosapent ethyl (IPE), and omega-3 (DHA/EPA) [13].

**Metformin**. Metformin is the most common drug prescribed for type 2 diabetes and was originally derived from galegine (a guanidine derivative found in the plant *Galega officinalis*). This compound reduces glucose production and increases insulin sensitivity. The mechanism of action is linked to the activation of the energy sensor AMPK, but other signaling pathways such as mTOR, eEF2K, TGF-β, NFAT, MAPK, and mitochondrial pathways of ROS production, NOX, and MnSOD are involved, with direct effects on metabolic homeostasis, glucose use, and cellular stress responses [16]. Metformin shows neuroprotective effects by reducing oxidative stress, inhibiting the permeability transition pore, and restoring mitochondrial membrane potential [17]. A pilot randomized clinical trial of metformin (1000 mg twice a day for 12 months) in elderly subjects with MCI indicated that the treatment was tolerated but had no significant effects on cognitive measures (ADAS-cog score), CSF biomarkers, or cerebral glucose metabolism (PET scans) [18]. A phase II/III trial is active to assess long-actin metformin in nondiabetic, overweight, or obese subjects with MCI or early AD (clinicaltrials.gov: NCT04098666). The treatment course includes 500–2000 mg/d of metformin for up to 24 months and evaluation of cognitive performance tests, MRI scans, and plasma biomarkers (Aβ, tau, NfL). A posthoc analysis identified changes in genome-wide DNA methylation in patients treated with metformin. Several pathways related to mental abilities (delirium) showed methylation changes, including genes involved in longevity, glutamatergic synapse activity, AMPK signaling, circadian entrainment, cholinergic synapse activity, mTOR signaling, and glucose metabolism/transport [19].

**Semaglutide**. It is a glucagon-like peptide-1 (GLP-1) agonist prescribed for type 2 diabetes in subcutaneous and oral dosage forms. In the brain, GLP-1 is neuroprotective and involved in insulin signaling, cognition, learning, synaptic transmission, and neuronal death [20]. The activity of semaglutide occurs by promoting insulin secretion, inhibiting glucagon release, and suppressing gluconeogenesis. The therapeutic potential of semaglutide has been established with clinical trials for obesity, diabetes, nonalcoholic steatohepatitis, and neurodegenerative disorders such as Parkinson’s and Alzheimer’s disease [21]. The neuroprotective effect of semaglutide occurs through enhancement of autophagy, inhibition of apoptosis, and blocking Aβ cytotoxicity, as observed in cell and animal AD models [22]. Furthermore, in PD mice models, semaglutide treatment showed neuroprotective effects, including rescue of compromised motor neurons, reduction of inflammatory responses, reduced lipid peroxidation, increased autophagy, protection of dopaminergic neurons, and reduction of α-syn accumulation [23,24]. The pharmaceutical company Novo Nordisk is performing phase III trials to test its oral drug semaglutide (commercial name: Rybelsus) for treating AD [25]. These studies are recruiting more than 3700 patients with early AD; the trials (clinicaltrials.gov: NCT04777409 and NCT04777396) will be with oral semaglutide (14 mg/d for up to 173 weeks) in elderly AD patients (55–85 years) with primary outcome measures of the cognitive test (CDR-SB score) and secondary outcome measures of daily living scales (ADCS-ADLMCI), cognition (ADAS-Cog, MoCA, MMSE), and CSF biomarkers. Semaglutide is also studied in a phase II trial for PD, and the participants receive 1.0 mg/week of semaglutide (subcutaneous) for a period of 48 months (clinicaltrials.gov: NCT03659682). The study will measure the effects of the treatment on motor symptoms, nigrostriatal degeneration, cognitive function, quality of life, and non-motor symptoms of PD. Additionally, plasma and CSF biomarkers will be quantified after 12, 24, 36, and 48 months of the study.

**Liraglutide**. Liraglutide is a GLP-1 analog also used to treat diabetes and has a similar mechanism of action to semaglutide. In APP/PS1 mice, the treatment with liraglutide (25 nmol/kg daily for 8-weeks) prevented memory impairment, synapse loss, and deterioration of synaptic plasticity in the hippocampus. Remarkably, the deposition of Aβ was significantly reduced (40–50%) with lower neuroinflammation signs [26,27]. In the MPTP mice model of PD, the administration of liraglutide showed neuroprotective effects, preventing neuronal impairment and reducing apoptosis and inflammatory cytokines [28,29]. A pilot trial with AD patients treated with liraglutide (1.8 mg daily for 6-months) showed no significant effect in Aβ deposition or in cognitive measures (clinicaltrials.gov: NCT01469351) [30]. Additionally, liraglutide prevented the decline of brain glucose metabolism in AD patients (measured by PET scans) [31]. In nondiabetic subjects, liraglutide administration (12 weeks) showed improvement in neuronal connectivity in the hippocampus and anterior medial frontal structures [32]. A phase II clinical trial of liraglutide (1.8 mg daily, subcutaneous injections for 12 months) evaluated changes in cerebral glucose metabolic rate (MRI volume scans) and cognitive measures (clinicaltrials.gov: NCT01843075) [33]. The study did not reach the primary outcome with no significant differences in glucose metabolic rate, but the liraglutide group showed improvements in cognitive tests and lower brain volume loss in comparison with the placebo group. The phase II trial with PD patients indicated that liraglutide (1.2–1.8 mg daily for up to 14 months) improved non-motor function and daily life activities (clinicaltrials.gov: NCT02953665). These results indicate the potential of liraglutide to improve some critical signs and symptoms of PD.

**Insulin**. Impaired mitochondrial metabolism is present in AD, particularly due to abnormal insulin signaling and insulin resistance. Insulin is a peptide secreted by the pancreas and plays a critical role in the regulation of glucose metabolism [34] and critical roles in mitochondrial dynamics, mitochondrial biogenesis, and mitophagy [35]. However, the neuronal responses to insulin are defective in AD; this is correlated with alterations in the cerebral insulin receptor, particularly the long isoform INSRα-B that is concentrated in microvessels rather than in the parenchyma neurons, and also with significantly lower concentrations of insulin receptors in the parietal cortex in AD subjects [36]. Furthermore, the transport rate of insulin across the BBB is very low (<0.03 μL/g·s), which in conjunction leads to insulin resistance in AD and worse cognitive measures. A reduced activity of insulin/insulin-like growth factor signaling (mediated by the insulin receptor substrate 1 gene, *Irs1*) in the brain is linked with increased energy expenditure, locomotion, and insulin sensitivity [37]. This is critical for brain function because the loss of *Irs1* causes age-dependent mitochondrial dysfunction, metabolic stress, and reflected systemic effects in peripheral tissues. Modulation and improvement of bioavailable insulin in the brain or restoring insulin sensitivity could prevent, delay, or treat neurodegeneration. To overcome this metabolic bottleneck, several clinical trials of insulin and insulin-related drugs have been performed in subjects with MCI/AD to understand the impact of insulin signaling in the brain and assess the efficacy of diabetes drugs for the treatment of neurodegenerative disorders.

Intranasal insulin is administered instead of peripheral insulin to promote direct entry to the brain through the bulk flow, olfactory nerve channels, and trigeminal perivascular channels, avoiding the BBB limitations and potential hypoglycemia [34]. Intranasal insulin (4 × 40 IU/d for 8-weeks) improved memory, attention, and mood in healthy subjects without perceivable side effects [38,39]. Pilot studies with MCI or early AD patients indicate that intranasal insulin improved cognition and Aβ_40/42_ ratio in plasma; the improvement was only observed in APOE-e4 carriers [40,41]. The insulin treatment also correlated with preserved brain volume (MRI) and a reduction of the tau-P181/Aβ42 ratio in subjects with MCI or mild-to-moderate AD [42]. A randomized controlled trial (clinicaltrials.gov: NCT0043868) showed that nasal insulin (20–40 UI for 4 months) preserved cognitive measures (ADAS-Cog) and cerebral glucose metabolism (PET imaging) in subjects with MCI or mild-to-moderate AD [43]. Phase II/III trials continued to examine the feasibility, safety, and efficacy of intranasal insulin (40 UI daily for 12 months) for the treatment of MCI or early AD (clinicaltrials.gov: NCT01767909). However, no differences were observed between insulin treatment and controls, as measured by cognitive tests (ADAS-Cog), CSF biomarkers, or MRI scans [44]. Additional clinical trials of intranasal insulin (clinicaltrials.gov: NCT05006599) or combination intranasal insulin/empagliflozin (clinicaltrials.gov: NCT05081219) are currently ongoing to confirm the efficacy of the treatment to decrease glucose resistance, improve neuronal bioenergetics, and increase insulin signaling in the brain [13].

Instead of administering insulin directly, some drugs with the activity of insulin sensitizers are used as interventions to re-establish insulin resistance and improve mitochondrial glucose metabolism in the brain. These compounds are widely used for diabetes and tested for their potential efficacy in treating neurogenerative disorders [45].

**Dapagliflozin**. This antidiabetic drug is an inhibitor of the sodium-glucose cotransporter 1 (SGLT2), blocking glucose reabsorption in the kidney [45]. Dapagliflozin exerted mitochondrial protection, preventing swelling and normalized mitochondria size in diabetic mice (C57BL/6NCrl), also significantly reducing lipid peroxidation and normalization of respiratory ratio [46]. In diabetic mice, dapagliflozin showed metabolic and neuroprotective effects [47], by reducing mitochondrial dysfunction, inflammation, and apoptosis [48]. A prospective study of antidiabetic medications indicated that the use of metformin, GLP-1 analogs, and SGLT2 was associated with a lower risk for dementia [49]. A phase I/II trial in subjects with probable AD tested the effects of 10 mg dapaglifozin (daily for 12 weeks) (clinicaltrials.gov: NCT03801642). The primary outcome is cerebral N-acetyl aspartate (NAA) levels using MRI; this compound is produced by mitochondria and is reduced in neurodegeneration. Furthermore, the participants are examined for brain glucose metabolism (PET) and plasma biomarkers. The trial was concluded in April 2022, but the findings have not been published. A pilot study of dapagliflozin on mitochondrial dysfunction and impaired insulin signaling/action (10 mg/day for 2 weeks) (clinicaltrials.gov: NCT01439854). Overall, dapagliflozin treatment reduced plasma glucose and increased insulin-stimulated disposal by 36%, a reduction of basal glucose oxidation, increased lipid oxidation, reduced ATP synthesis, and increased plasma ketones were correlated with dapagliflozin administration [50].

**Pioglitazone**. This is an oral antidiabetic drug from the thiazolidinedione class, that is FDA-approved for treating type 2 diabetes mellitus in adults. At the molecular level, pioglitazone activates the peroxisome proliferator-activated receptor (PPAR-γ), promoting energy production, glucose metabolism, and lipid metabolism. Furthermore, this drug inhibits mitochondrial oxidative stress and inflammation and improves mitochondrial biogenesis, dynamics, and function by activating the PPAR-γ/PGC-1α signaling pathway [51]. The treatment with pioglitazone (12.5, 25, and 50 μg/mL) may exert some side effects, such as the generation of ROS, reduction of mitochondrial membrane potential, mitochondrial swelling, and release of cytochrome C, as observed in mitochondria isolated from the brain and heart of Wistar rats [52]. An analysis of health insurance records discovered that diabetic patients who received pioglitazone had a lower risk of dementia (decreased by 23%) during the 5-year follow-up period. This clinical study concluded that pioglitazone showed a protective effect against dementia in diabetic patients, with a time- and dose-dependent trend in comparison to controls [53]. Another prospective study with subjects ≥ 60 years of age also observed that long-term use of pioglitazone was associated with a lower risk of dementia (47% reduction) relative to nondiabetic subjects and that diabetic patients without pioglitazone had a 23% decrease in dementia risk [54]. The effect of pioglitazone use on dementia in consideration of stroke or ischemic heart disease occurrence was examined in a 15-year follow-up cohort study. Overall, pioglitazone showed a 43–54% reduction in dementia risk in comparison with non-treated patients [55]. These findings indicate the utility of personalized treatments for diabetic patients to reduce the incidence of neurodegenerative diseases [56]. The phase II clinical trial of pioglitazone (15 mg daily at 1-week intervals for 18 months) shows that it is safe and well tolerated in AD patients (clinicaltrails.gov: NCT00982202) [57]. The phase II trials of pioglitazone in MCI/AD (clinicaltrails.gov: NCT01931566 and NCT02284906) were terminated in 2019 due to the lack of efficacy of the drug or significant effects on cognition.

**Tricaprilin**. It is an oral formulation of caprylic triglyceride, administered as a supplement or medical food. Formulations of tricaprilin may improve mitochondrial activity and increase ketone bodies, regulating the abnormally low rates of cerebral glucose metabolism in AD patients, as observed in APOE4 carriers [58,59]. After administration, tricaprilin is hydrolyzed to octanoic acid and then metabolized to ketones to be used as an alternative energy substrate in the brain [60]. The formulation of caprylic triglyceride, also known as AC-12-010, was administered daily (40 g powder mixed with 4–8 oz of water) for 26 weeks in APOE4 non-carrier subjects with a diagnosis of mild-to-moderate AD. The phase II/III trial (clinicaltrials.gov: NCT01741194) showed that the compound was safe and well-tolerated, but it failed to improve cognition or functional abilities (measured with the ADAS-Cog11 and AD-CGIG tests) [61]. Another phase III trial of tricaprilin is active to evaluate its safety and efficacy among subjects with mild-to-moderate AD (clinicaltrials.gov: NCT04187547).

**Hydralazine**. This FDA-approved drug (hypertension medication) has been shown to increase mitochondrial activity and respiration, antioxidant properties through the Nrf2 pathway, and activation of autophagy [62]. Furthermore, hydralazine reduced Aβ misfolding and oxidative lipid damage in PC12 cells [63] and inhibited Aβ aggregation, glycation, and reduction of neurotoxic effects (Neuro2a cells) [64]. The phase III clinical trial is currently assessing the efficacy of hydralazine in early-stage AD patients who take one of the acetylcholinesterase inhibitors (AChEI), donepezil, rivastigmine, or galantamine (clinicaltrials.gov: NCT04842552). The participants will receive hydralazine tablets (25 mg) every eight hours for one year to determine the effects on cognitive and functional measures. This trial is recruiting participants, and the outcomes will be available in the upcoming years.

**Icosapent ethyl (IPE)**. IPE, also known as ethyl eicosapentaenoic acid (E-EPA) or vascepa, is a purified omega-3 fat from fish oil. Oral administration of EPA changes mitochondrial lipid composition, preserving mitochondrial fusion protein OPA-1 and the activity of oxidative phosphorylation, overall reducing mitochondrial damage [65]. A pilot study of ethyl-EPA in AD patients showed only a minimal difference between treated and control groups after 12-weeks of treatment (500 mg twice daily), with no perceivable effects in cognition [66]. A phase II/III clinical trial of IPE is evaluating the efficacy of this purified form of omega-3 fatty acid eicosapentaenoic acid (clinicaltrials.gov: NCT02719327). The study is active but not recruiting participants; it is expected to analyze the effects on brain blood flow, AD biomarkers, and cognitive performance of AD patients 950–75 years), with a dose of 4 g daily for up to 18 months.

**Omega 3 (DHA/EPA)**. Fatty acids from fish oil and seaweed are enriched in docosahexaenoic acid (DHA) and eicosapentaenoic acid (EPA). Several properties have been attributed to DHA/EPA, including antioxidant, antiinflammation and neuroprotective. Supplementation with omega-3 causes alteration in mitochondrial membrane composition, respiration kinetics, redox status, and mitochondrial biogenesis [67,68]. Administration of omega-3 supplements to patients with AD (1.7 g DHA + 0.6 g EPA) showed no significant differences in oxidative stress biomarkers [69]. Phase III trial of DHA supplementation in individuals with mild-to-moderate AD indicated that the 2 g/d doses had no beneficial effects on cognitive decline after 18 months of treatment (clinicaltrials.gov: NCT00440050) [70]. Long-term supplementation of omega-3 (800 mg DHA + 225 mg EPA) showed similar outcomes, with no effects on prevention or delay of cognitive decline in AD (clinicaltrials.gov: NCT00672685) [71]. Finally, there is another phase III trial of EPA/DHA currently recruiting 400 participants with AD (clinicaltrials.gov: NCT03691519). The study will test the effects of a 3 g DHA/EPA supplement for a period of 18 months, and the participants will be evaluated in cognitive performance and mental-verbal fluency.

**Urolithin A (UA)**. This metabolite is produced by gut microflora from foods rich in ellagitannis (a class of polyphenols found in pomegranate, walnuts, and berries), but UA can be directly supplemented by patients to overcome bioavailability limitations [72]. UA promotes mitophagy and improves mitochondrial function in pre-clinical models of aging and in elderly humans [73]. In APP/PS1 mice, supplementation with UA ameliorated cognitive impairment, reduced neuronal death, neuroinflammation, and Aβ deposition [74]. Furthermore, the bioactive compound enhanced neurogenesis and reduced microgliosis and astrocytosis in the cortex and hippocampus of mice. The molecular mechanism of UA is not completely clear, but it is related to the activation of AMPK, decreasing proinflammatory cytokine levels (P65NF-κB and P38MAPK), BACE1 inhibition, and promoting AβPP degradation, overall promoting neuronal protection and showing potential to treat neurodegeneration. UA may activate and promote mitophagy, with neuroprotective properties through the reduction of inflammation responses [75]. In different in vitro and in vivo models of AD, the supplementation of pomegranate extract or purified urolithin A/B showed significant antioxidant properties, anti-inflammatory properties, and inhibition of Aβ learning and memory deficiency in transgenic mice. Moreover, this nutraceutical compound is proposed as an alternative or complementary treatment of AD because of its ability to reduce BACE1 activity, Aβ deposition, ROS production, inflammation, and restore or induce mitophagy.

Therapeutics targeting mitochondrial dysfunction in PD aim to promote the clearance of damaged mitochondria or restore mitochondrial homeostasis in dopaminergic neurons [6]. Of the active trials registered for PD, none of phase III is focused on energy, mitochondria, or antioxidants; the vast majority of the compounds are designed for dopaminergic symptom relief. In phase II, there are two compounds with mechanisms of action in energy and mitochondria: nicotinamide riboside and terazosin, and three more as antioxidants: deferiprone, sulforaphane, and tocovid suprabio [14].

**Nicotinamide riboside**. This compound is a different form of vitamin B3, which in the brain promotes the formation of NAD+, which usually declines with aging and neurodegeneration. A phase I clinical trial studied the effects of nicotinamide riboside (NR) oral intake (1000 mg for 30 days) in individuals with PD (clinicaltrials.gov: NCT03816020). Overall, NR treatment was well tolerated, promoting increased NAD levels in the brain (MRI scan and CSF) and increased brain glucose metabolism [76]. The clinical benefits were associated with increased NAD levels and increased mitochondrial activity. This study advanced to phase II where the PD patients will take NR for up to 1 year; the results of this study are expected in 2024.

**Terazosin**. This is an alpha-blocker used as an antihypertensive drug and for urinary retention. The mechanism of action of terazosin involves binding to phosphoglycerate kinase 1, increasing its activity, enhancing glycolysis, and increasing ATP levels in the brain to overcome impaired energy metabolism. The phase I clinical trial assessed terazosin (5 mg for 12-weeks) in PD patients. The participants showed a significant increase in ATP levels (brain and blood), but with some mild side effects such as dizziness and lightheadedness [77]. The study is advancing to phase II (clinicaltrials.gov: NCT03905811).

**Deferiprone**. Alterations in iron transport and storage are characteristic of PD and other neurological disorders. This condition leads to increased oxidative stress, protein aggregation, lipid peroxidation, and mitochondrial dysfunction [78]. As a result, the use of the iron chelator deferiprone was proposed to reduce nigrostriatal iron content in PD. The phase II clinical trial of deferiprone was conducted to assess the effectiveness of a dose of oral medication (15 mg/kg) for 36 weeks. Unfortunately, the administration of deferiprone was associated with worse scores in PD symptomatology in comparison with placebo, even though the nigrostriatal iron content decreased more in the deferiprone group (clinicaltrials.gov: NCT02655315) [79]. These results make it unlikely that additional trials will be conducted for deferiprone due to the lack of effectiveness in the treatment of PD.

**Sulforaphane**. Sulforaphane is a phytocompound found in seeds and cruciferous vegetables (broccoli, brussels sprouts, and cabbage). Different properties are attributed to sulforaphane, including cytoprotective, antioxidant, anti-inflammatory, and modulator of mitochondrial functions and dynamics [80,81]. Sulforaphane supplementation will be studied in addition to existing PD treatment (24 weeks) to determine its effects on cognitive function, motor symptoms, and PD biomarkers (clinicaltrials.gov: NCT05084365).

**Tocovid suprabio**. This is a formulation of vitamin E tocotrienols with potential therapeutic use in PD for delaying motor dysfunctions. Tocotrienols have neuroprotective effects in in vitro and in vivo models of PD [82]. The phase II trial will evaluate the effects of a tocotrienol formulation (HOV-12020 palm oil-derived vitamin E) with a dosage of 400 mg/day for 12 months. The expected outcomes include the progression of cognitive impairment, motor and non-motor outcomes, and progression of PD biomarkers (clinicaltrials.gov: NCT04491383). Currently, the trial is in the recruitment phase to select 100 participants; results are expected in a couple of years.

**PINK1 activators**. Mutations in the protein ubiquitin kinase PINK1 may lead to defective mitophagy in early-onset PD. PINK1 and the ubiquitin E3 ligase Parkin work together in mitophagy processes; defective versions of these proteins are unable to clear dysfunctional mitochondria, leading to a progressive loss of dopaminergic neurons and the progression of motor symptoms in PD [83]. Furthermore, the deposition of α-syn induces mitochondrial dysfunction and alterations in mitophagy, causing PINK1 accumulation in neurons [84]. Several small-molecule PINK1 activators have been discovered and tested as potential treatments for PD [85].

**MTK458** is a molecule that targets PINK1, which promotes mitophagy. In cell and animal PD models, the treatment with MTK458 helped to clear α-syn and reduce pS65-Ubiquitin, the substrate of PINK1 [84]. Confirming that activation of PINK1 is a viable therapeutic alternative for disease modification in PD.

## 4. Mitochondrial Transference

**Figure 4:**
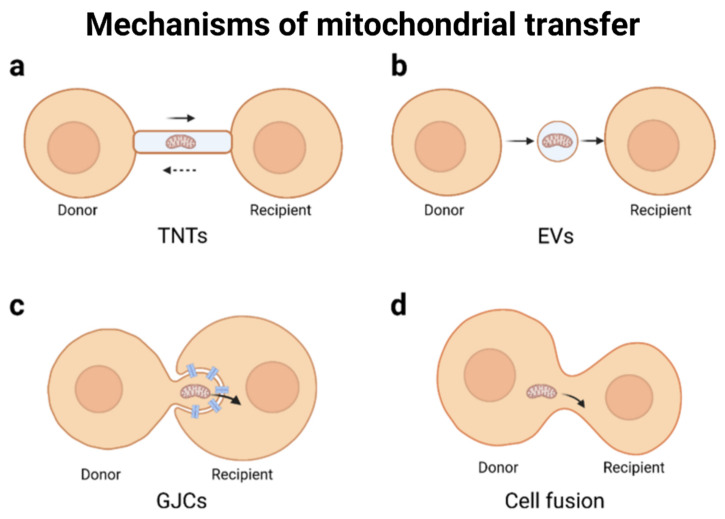
Mechanisms of mitochondrial transfer. A simplified visualization of intercellular mitochondrial transfer modes. (**a**) Mitochondria are transported between cells via the TNT structure, and this transport is bidirectional. (**b**) The mitochondria-containing EVs bud off/are secreted from the donor cells and then uptake by recipient cells. (**c**) The mode of GJC-mediated mitochondrial transfer is ambiguous. Nonclassic form AGJs are plausible: mitochondria exist in the protrusions of donor cells, and the recipient cells connect to the donor cells through invaginating GJCs. Then the recipient cell internalizes GJCs and absorbs mitochondria. (**d**) Cell fusion can be spontaneous or artificial. Reproduced with permission from Liu Z, et. al., Cell Biol 2022, (66): 13578 [88].

In neurons, mitochondria exhibit more functions than energy (ATP) production; these organelles also regulate axonal and dendritic development, axonal regeneration, lipid metabolism, the production of antioxidants, ROS regulation, calcium homeostasis, and synaptic functions [86]. Furthermore, glial mitochondria provide bioactive metabolites to neurons. As a consequence, mitochondrial dysfunction and altered dynamics may have a direct impact on neurodevelopment, neural circuit development, pathophysiological conditions, aging, and neurodegeneration. Mitochondria are known to be highly dynamic, changing morphology, cellular location, and distribution. When mitochondria are damaged, they are removed and delivered to lysosomes for degradation through the process of mitophagy. Normally, this process of mitophagy occurs within the same cell, indicating an internally regulated process to recycle its own compromised mitochondria. However, neurons can release damaged mitochondria to be degraded by glial (astrocytes) cells, indicating a complex and dynamic transcellular system for the degradation of axonal mitochondria [87]. On the contrary, the idea of transferring or transplanting healthy mitochondria into compromised cells may serve to regain or rescue normal respiration functions and promote the survival of the recipient cells. The phenomenon of mitochondrial transfer has been observed under different physiological and pathological conditions. Intercellular mitochondrial transfer occurs through tunneling nanotubes, extracellular vesicles, and gap junction channels [88] (Figure 4).

In a mice model of intracerebral hemorrhage (ICH), the intravenous transplantation of functional mitochondria from astrocytes into neurons promoted the recovery of superoxide dismutase activity, reduced neurological deficits, and reduced oxidative stress (ROS) and neuronal death [89]. Furthermore, this strategy promoted neurite extension, overexpression of the synaptogenesis pathway, and neuroplasticity, confirming the utility of mitochondrial transfer as a treatment to recover brain functions after ICH. Likewise, astrocyte-derived mitochondria are incorporated by microglia, producing bioactive humanin peptide and inducing expression of superoxide dismutase [90]. The treated microglia showed enhanced phagocytic activity and reduced proinflammatory responses from ICH.

In a mice model of ischemia, the transference of healthy mitochondria from astrocytes to adjacent neurons after stroke promoted cell survival [91]. The mechanism of mitochondrial release is mediated by calcium, CD38, and cyclic ADP ribose, and blocking these signaling pathways causes worsening of neurological symptoms. The transference occurred through extracellular vesicles (300–1000 nm in size) containing functional mitochondria derived from cortical astrocytes. These observations indicate a dynamic cell-to-cell signaling mechanism among different cell types in the brain and the potential of mitochondrial transference as a neuroprotective treatment. The treatment with mitochondria isolated from rat primary astrocytes enhanced cell viability and restored hydrogen-peroxide-damaged neurons (ischemic stroke), confirming that mitochondria transfer therapy can treat neurological disorders [92]. Interestingly, mild hypothermia (33 °C) seems to facilitate the mitochondrial transfer or maintain mitochondria in a functional state into the extracellular medium, in comparison to physiological temperature (37 °C). As observed in the experiments of mitochondria transfer from astrocytes in hypothermia to injured neurons during oxygen-glucose deprivation/reoxygenation [93].

In a mice model of AD, the intravenous transfer of fresh human-isolated mitochondria showed reduced cognitive deficits, neuronal loss, and gliosis in the hippocampus [94]. The beneficial effects are related to increased citrate-synthase and cytochrome-c oxidase activities (mainly in the liver) in treated mice in comparison with the untreated group. The direct injection of mitochondria into the medial forebrain bundle of PD rats showed enhanced mitochondrial function in the substantia nigra neurons, resistance to oxidative stress and neuronal death, and even signs of neurite outgrowth [95]. Three months after the mitochondrial transference treatment (allogenic and xenogeneic), the treated PD rats showed improved locomotive activity, a decrease in dopaminergic neuronal loss, and restored mitochondrial dynamics [95]. Mitochondria isolated from human hepatoma cells enter human neuroblastoma cells (SYSY5Y) and PD mice (intravenously) without any apparent side effects [96]. Remarkably, the transferred mitochondria were distributed into the brain, liver, kidney, muscle, and heart, promoting increased mitochondrial activity (respiration), endurance, decreased ROS levels, and preventing cell death (apoptosis and necrosis). Intranasal infusion of allogeneic mitochondria in PD rats indicated the feasibility of delivering mitochondria intranasally and bypassing the blood-brain barrier [97]. The treated rats showed a significant improvement in rotational and locomotor behavior, neuronal survival, recovery from lesions of the substantia nigra, and restored mitochondrial function. The experiments indicated that the mitochondria entered through the accessory olfactory bulb and doublecortin-positive neurons of the rostral migratory system and were then located in both cerebral hemispheres. Human iPSC-derived astrocytes rescued injured dopaminergic neurons by transferring functional mitochondria [98]. The spontaneous release of functional mitochondria from astrocytes into the media allowed their internalization by injured neurons via a phospo-p38-dependent pathway. The specific signaling cross-talk between the donor and the receptor cells is not known but could be related to the initial release of injured mitochondria by neurons as a stress signal, which then mediates a cellular response of glia cells to promote intercellular mitochondrial transfer to heal the injury or lesion in the brain [99]. Overall, mitochondrial transference offers an opportunity to develop novel therapeutic strategies for neurological disorders.

## 5. Perspectives on Mitochondrial Therapies for Neurodegeneration

**Drug pipeline in clinical trials**. There are 82 agents in phase II clinical trials for AD, but only four are related to metabolism and bioenergetics (dapagliflozin, intranasal insulin, intranasal insulin + empagliflozin, T3D-959 a PPARδ/γ agonist, and a supplement of N-acetylcysteine/L-carnitine tartrate/nicotinamide riboside/serine), and one agent for oxidative stress (omega 3, DHA). Whereas there are 30 agents in phase I clinical trials for AD, and only one is focused on metabolism and bioenergetics (caprylic triglyceride) [13]. In the case of PD agents currently under clinical trials, there are 74 in phase II and 22 more in phase I [14]. In these phase II clinical trials, only two agents are focused on energy and mitochondria (nicotinamide riboside and terazosin), and three more are antioxidants (deferiprone, sulforaphane, and tocovid suprabio). In early phase I, no therapeutic compounds target mitochondria, energy, or antioxidants. The findings of these phase II/I clinical trials will be available in the upcoming years; if successful, they will advance to phase III to confirm effectiveness in the treatment of neurological disorders.

**Figure 5:**
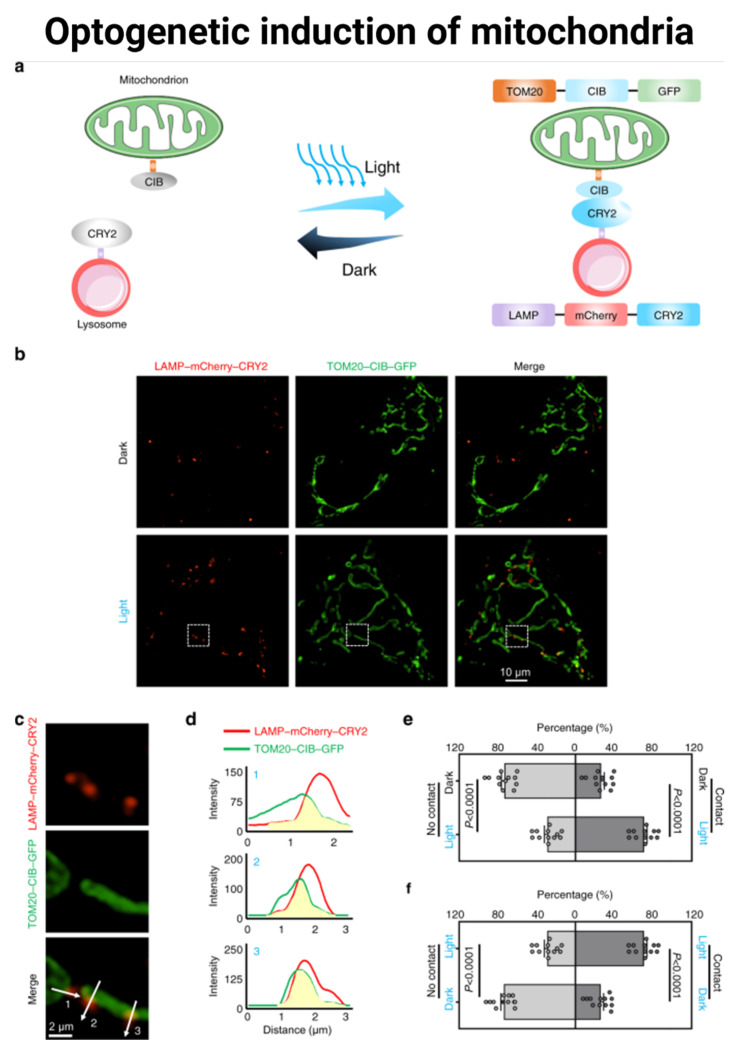
Optogenetics can be used to induce mitochondria-lysosome contacts. (**a**) Schematic representation of optogenetic induction of MLCs. Light-sensitive proteins CRY2 and CIB are anchored to lysosomes and mitochondria via the specific organelle-targeting transmembrane domains LAMP and TOM20, respectively. Blue light illumination induces CRY2-CIB association and facilitates the formation of MLCs. GFP and mCherry are used as expression markers. (**b**) Representative structured illumination microscopic images of mitochondria (green) and lysosomes (red) with or without blue light exposure for 20 min at 300 μW/cm^2^. (**c**) Partially enlarged images of Figure 5b. (**d**) Intensities of GFP and mCherry on the white arrows in Figure 5c. (**e**) Quantification of percentages of lysosomes contacting and not contacting mitochondria without or with blue light exposure. (**f**) Quantification of percentages of lysosomes contacting and not contacting mitochondria with blue light exposure or with blue light exposure followed by dark for 24 h. For (**e**,**f**), n = 12 cells examined over 3 independent experiments. Data are presented as M ± SEM. The statistical differences between the experimental groups were analyzed by double-tailed Student’s *t* test. When *p* < 0.05, it was considered to have statistical significance. Reproduced with permission from Qiu K, et al., Nat Commun 2022, 13: 4303 [100].

**Optogenetic control of mitochondria**. Optogenetics allows the direct manipulation of some cellular functions with light stimulation. A blue-light induction via optogenetic control was able to induce mitochondrial fission and control mitochondria-lysosome contacts. Remarkably, this optogenetic strategy partially restored the mitochondrial functions of SLC25A46^−/−^ cells, such as defects in mitochondrial fission and hyperfused mitochondria [100] (Figure 5). Optogenetics allowed the targeting of the heterologous channelrhodopsin-2 fusion protein to the inner mitochondrial membrane [101]. This caused a light-induced selective change in mitochondrial membrane potential, depolarization, and mitochondrial autophagy. Furthermore, optogenetic-mediated mitochondrial depolarization can control the cell fate decision toward apoptosis (under sustained moderate light illumination) or cytoprotective effects (mild light illumination). A light-dependent control of the mitochondrial membrane potential allows modulation of ATP production, Ca^2+^ uptake/release, substrate accumulation, and respiratory metabolism [102]. This molecular strategy efficiently modulates mitochondrial functions in specific tissue regions and even with single-cell resolution. The mitochondrial inner membrane protonmotive force (PMF) was precisely controlled through an engineered mitochondrial-targeted light-activated proton pump [103]. The photoactivation increased PMF in a dose-dependent manner, allowing ATP synthesis and mitochondrial resistance. Additionally, the transient activation allowed the cells to adapt to hypoxic events and modulate metabolism.

Light stimulation has been proven to rescue memory impairment in AD and has some neuroprotective effects in animal models [104]. Whereas deep brain stimulation-guided optogenetics rescues PD symptoms [105]. It is expected that optogenetic light stimulation strategies will advance to clinical applications as alternative therapeutic approaches for neurological conditions. The optogenetic tools have been expanded to control the cellular metabolism and dynamic changes in mitochondria to overcome metabolic alterations in cardiovascular disease, cancer, obesity, and neurological disorders [106]. Photobiomodulation with laser stimulation showed positive age-related effects in aged mice, promoting recovery of cytochrome c oxidase levels; this enzyme is important and responsible for oxygen consumption in the mitochondrial electron transport chain [107]. Furthermore, photobiomodulation showed anti-inflammatory properties, resulting in a promising strategy to treat brain pathological disorders [108].

Coupling with the novel optogenetic molecular tools for induction of mitochondria, the light stimulation could be targeted to specific brain regions or cell populations to target mitochondrial dysfunction.

**Mitochondrial gene editing.** Mutations in mitochondrial DNA (mtDNA) are related to several diseases, but not all mitochondria carry mutations, leading to a heteroplasmy state where normal and altered mtDNA coexist in the same cell population [109]. Novel methods of gene editing focus on the modification of mtDNA, including the use of mitochondria-targeted nucleases (mitoNucleases) and single-point mutations, to reverse abnormal mutations and alleviate clinical phenotypes. The delivery of mitochondrial gene editing tools occurs through adenovirus, lentivirus, retrovirus, or adeno-associated virus, allowing specific targeting of disease-relevant tissues. Dopaminergic neurons in a mouse model of PD showed striatal dysfunctions associated with mitochondrial DNA defects [110], but the accumulation of mitochondrial mutations in neurons is mainly related to aging [111]. The technology is still in its early stages, but results are promising for the design of selective or specific molecular tools to overcome mitochondrial damage in aging and neurological disorders.

Editing the mitochondrial genome can be performed with mitochondria-targeted zins-finger nucleases/deaminases (mitoZFNs) or mitochondria-targeted transcription activator-like effector nucleases (mitoTALENs). Use of mitoZFN is useful for C-to-T base conversions without inducing additional insertions or deletions in human cells, with an efficiency of 60% in nuclear DNA and 30% in mtDNA [112]. mitoTALENs cleave specific sequences of the mtDNA to remove pathogenic mutations. Designed mitoTALENs were able to correct mtDNA point mutations related to myoclonic epilepsy and Leigh syndrome; these changes improved respiratory capacity and oxidative phosphorylation activity [113]. Additional molecular tools based on mitoZFNs and mitoTALENs are currently being tested for precision mitochondrial DNA editing with high fidelity. For example, the use of bacterial toxin DddA-derived cytosine base editors (DdCBEs) composed of split DddA_tox_ cytosine deaminase, custom-designed TALE DNA-binding proteins, and a uracyl glycosylase inhibitor to enable editing of mtDNA in human cells for therapeutic purposes [114]. Furthermore, the development of compact zinc finger DdCBEs with improved performance and their delivery through engineered AAV9 vectors to efficiently correct disease-associated mitochondrial mutations [115].

**CRISPR/Cas system**. The modification of the mitochondrial genome using the CRISPR-Cas9 system is still challenging, mainly due to the low efficiency of delivering the molecular components into the mitochondria. However, recent efforts have used the NADH-ubiquinone oxidoreductase chain 4 (ND4) and the RNA transport-derived stem-loop element (RP-loop) to improve the efficiency of mtDNA modification, demonstrating the potential use of this system to target mitochondrial diseases [116]. Furthermore, the CRISPR/Cas12a system has been adapted to target mitochondrial material (mitoCRISPR/Cas12a), allowing mtDNA manipulation in mammalian cells [117]. A site-specific modification of the mtDNA of human cells was possible using the cytosine base editor Cas9-BE3 instead of regular Cas9, but still with low efficiency to deliver the gRNA into mitochondria [118]. In the same direction, the incorporation of transcription activator-like effector-linked deaminases (TALED) with the CRISPR/Cas system allowed a single point base conversion (A-to-G) in human mtDNA; the TALED can be custom-designed to improve efficiency (>49%) and specificity to target various mitochondrial genes [119]. Using an engineered CRISPR system allowed the induction of several insertion/deletion (InDel) events in mtDNA microhomologous regions, improving sgRNA multiplexing, and with the use of iniparib, a double-strand break repair inhibitor [120].

This platform is expanding, and recent efforts include genome-wide CRISPR to target mitochondria and genes related to mitochondrial stress responses (mTORC1 pathway) [121]. Screening with CRISPR/Cas identified mitochondrial genes and transcription factors related to metabolic resistance in cancer cells, allowing the identification of genes related to metabolic susceptibility and resistance that are relevant for different types of mitochondria-targeted therapies [122]. The CRISPR screening platform has been employed to identify mitochondrial components related to astrocyte-to-neuron conversion [123]. This comprehensive screening identified 150 significantly enriched mitochondrial proteins (including transporters, metabolic enzymes, and cell-type-specific antioxidants) that influence the reprogramming of brain cells. These recent advances in gene editing with CRISPR/Cas are now expanding to clinical and therapeutic applications, including mitochondrial genome editing as an innovative alternative to target mitochondria-related diseases [124].

## 6. Conclusions

For decades, the drug development pipeline for neurodegenerative diseases has been focused on two main mechanisms of action: neurotransmitters and protein aggregation. In fact, the handful of approved therapeutic compounds for Alzheimer’s and Parkinson’s disease are compounds intended to restore or maintain the brain levels of neurotransmitters or to remove abnormal protein aggregates. Remarkably, the observation of mitochondrial alterations and dysfunction in neurological disorders has been known for decades, but little effort has been established to develop specific therapies targeting these vital organelles or their molecular components. Here, we discussed the current findings of mitochondrial-related therapeutic compounds that aim to restore or maintain bioenergetics and functional antioxidant mechanisms in brain cells. Furthermore, we discussed alternative or innovative ideas that could be explored in mitochondrial therapies for neurodegenerative disorders, including optogenetics, gene editing, CRISPR/Cas engineering, and mitochondrial transference, which have demonstrated promising results in in vitro and in vivo models.

## Data Availability

Not applicable.
